# Exploration of genetic architecture through sib-ship reconstruction in advanced breeding population of *Eucalyptus nitens*

**DOI:** 10.1371/journal.pone.0185137

**Published:** 2017-09-22

**Authors:** Jaroslav Klápště, Mari Suontama, Emily Telfer, Natalie Graham, Charlie Low, Toby Stovold, Russel McKinley, Heidi Dungey

**Affiliations:** Scion (New Zealand Forest Research Institute Ltd.), 49 Sala Street, 3046 Rotorua, New Zealand; Technical University in Zvolen, SLOVAKIA

## Abstract

Accurate inference of relatedness between individuals in breeding population contributes to the precision of genetic parameter estimates, effectiveness of inbreeding management and the amount of genetic progress delivered from breeding programs. Pedigree reconstruction has been proven to be an efficient tool to correct pedigree errors and recover hidden relatedness in open pollinated progeny tests but the method can be limited by the lack of parental genotypes and the high proportion of alien pollen from outside the breeding population. Our study investigates the efficiency of sib-ship reconstruction in an advanced breeding population of *Eucalyptus nitens* with only partially tracked pedigree. The sib-ship reconstruction allowed the identification of selfs (4% of the sample) and the exploration of their potential effect on inbreeding depression in the traits studied. We detected signs of inbreeding depression in diameter at breast height and growth strain while no indications were observed in wood density, wood stiffness and tangential air-dry shrinkage. After the application of a corrected sib-ship relationship matrix, additive genetic variance and heritability were observed to increase where signs of inbreeding depression were initially detected. Conversely, the same genetic parameters for traits that appeared to be free of inbreeding depression decreased in size. It therefore appeared that greater genetic variance may be due, at least in part, to contributions from inbreeding in these studied populations rather than a removal of inbreeding as is traditionally thought.

## Introduction

Heritability is a measure of the proportion of phenotypic variance explained by genetic factors and is built on the principle of resemblance between relatives [1]. Narrow-sense heritability is therefore an important parameter for tree breeding programs as it represents the portion of variation that can be transmitted to the progeny. Accurate inference of the relationship among individuals in a breeding population is essential for reliable evaluation of genetic factors contributing to phenotypic variability. Relationship estimates are based on the probability that alleles from two randomly sampled individuals in the population are identical copies of recent single ancestral alleles (i.e. are identical by descent) [2] and estimated through path analysis [3] based on information from documented pedigrees. The level of relatedness and the connectedness through the pedigrees is defined by mating design and dictates the precision and amount of information which is potentially obtained from genetic tests [4]. This can be particularly relevant within the genus *Eucalyptus*, where degrees of relatedness can vary widely due to mixed mating system [5].

It can be difficult to establish test populations with precisely defined mating schemes due to technical or biological obstacles and instead, open-pollinated experiments are pursued [6]. Such experimental designs usually suffer from hidden (unaccounted for) relatedness [7] which produces a bias in the genetic parameters estimated in quantitative genetics analysis and misleading ranking of individuals to be selected for the next cycle of breeding [8–10]. Methods have been proposed to correct for hidden relatedness [11, 12], which are established on breeders’ estimates of selfing or inbreeding proportion based on previous experiences, rather than reality. Alternatively, it is possible to use a sire probability relationship matrix to define relationships by most probable mating events, and this has been successfully applied in animal breeding [13].

There are a number of algorithms developed for use in pedigree reconstruction [14–18] that have also been successfully applied in breeding [19], genetic conservation [20] or evolutionary studies [21, 22]. Pedigree reconstruction in open pollinated tests has been found to be a very effective tool for eliminating the adverse effect of hidden relatedness in quantitative genetics analysis [8–10, 23–27], especially in the initial phase of breeding programs where pedigrees are rather simple and lack connectivity. Additionally, the recovery of hidden relatedness enables the more efficient management of genetic diversity and prevents undesirable build-up of inbreeding in next generations of breeding populations. The selection of genetic markers, their type, quality and quantity is, however, crucial to obtain confident inference about relatedness [24] and improve accuracy in the genetic parameters [8, 28].

Our analysis is focused on evaluation of sib-ship reconstruction in a *Eucalyptus nitens* advanced breeding population under only partially tracked pedigree where only offspring genomic information was available. We investigated the effect of recovered relatedness (and inbreeding) on the precision of genetic parameter estimates in both univariate and multivariate genetic analysis. We also explored options to include an additional, non-additive (dominance) relationship matrix to improve breeding values accuracy, and to examine the impact of sib-ship reconstruction on the accuracy of genetic correlations and correspondence in ranking of breeding values and impact on genetic gain estimation.

## Materials and methods

### Material

**Fig 1:**
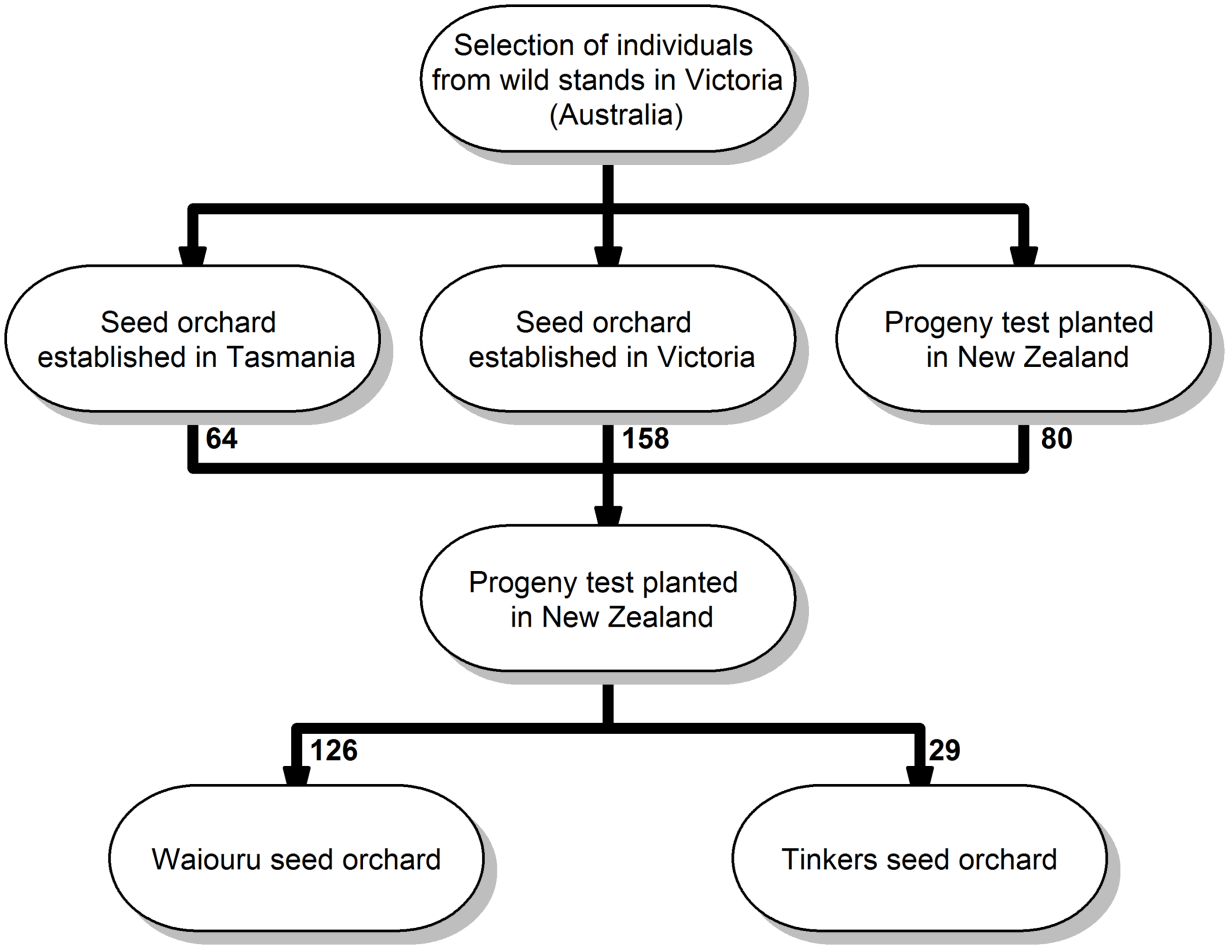
Breeding program history. History of the *E. nitens* breeding program established in New Zealand. The number of selected parents are given in the arrows between the different generations.

The *E. nitens* population in this study represented the latest generation of the breeding population [29]. The material was established as an open-pollinated (OP) test where families were established from two independently-sourced second generation seed orchards (Waiouru (46 OP families) and Tinkers (25 OP families)). Both seed orchards were established using forward selections from a progeny trial including material from three sources: Victorian provenances (showing the best growth [30]); from progeny trials at Rotoaira established in 1977 originating from two Australian breeding programs and; from progeny trials testing on New South Wales (NSW) provenances. The Waiouru seed orchard was originally designed as a clonal archive and included 123 individuals from the same number of families (123) (Fig 1).

Genomic DNA was extracted from the leaf tissue of 691 individual trees using a commercial NucleoSpin Plant II kit (Machery-Nagel, Duren, Germany) [31] and sent to GeneSeek, Inc. (a Neogene company, Lincoln, NE, USA) for genotyping [24] using the Illumina Infinium EUChip60K SNP chip [32]. SNP calling was performed based on the Maidenaria section reference. The marker data were filtered for genTrain score > 0.5, GenCall > 0.15, minor allele frequency (MAF) > 0.01, call rate > 0.6. Additionally, we applied the Hardy-Weinberg equilibrium (HWE) test (p-value > 0.05) to check for selective neutrality of the markers used.

Seven-year-old individuals within the open-pollinated progeny trial were phenotyped for the growth trait diameter at breast height (DBH [mm]) in winter of 2014. Wood quality was measured on two different log lengths—log 1 from 1.4-3m and log 2 from 3-6m during winter of 2015. Wood traits assessed include density (WD [kg/m3]), wood stiffness (log 1:ST1 [km/s]; log 2:ST2 [km/s]) and growth strain (log 1:GS1 [mm]; and log 2:GS2 [mm]) measured as log split width. Average tangential air-dry shrinkage was assessed only for log 2 (TS [%]). Wood density was measured as basic wood density through the maximum moisture content method [33]. Wood stiffness was measured indirectly using acoustic wave velocity [km/s] by using HITMAN (HM200). Growth strain was assessed by ripping logs with a chainsaw and and measuring the resulting openings at the end of the log (mm). Tangential air-dry shrinkage average was measured following standard wood quality assessment protocol [34].

### Sib-ship reconstruction

The sib-ship inference was obtained by implementing the algorithm described in Wang (2004). This algorithm assumes markers to be selectively neutral, unlinked between loci and in linkage equilibrium. Additionally, they should have two or more codominant alleles and meet the assumption of Mendelian segregation. The sib-ship reconstruction was performed following the above described algorithm implemented in the COLONY package [35] using 500 randomly selected markers that pass the filtering criteria. This number of genetic markers should be sufficient to reach the maximal assignment rate [36]. Markers were pre-checked for any pairwise linkage disequilibrium at less than 0.2 in terms of its composite estimate [37]. Since forest trees generally show large reproductive investment (production of both male and female flowers), the scenario was set under both polygamy and polyandry with the presence of inbreeding. The prior of maternal family size was set to 5 reflecting structure of pedigree and paternal family was set to 2 assuming some fathers contributing to more than one individual. Since both male and female flowers are present on the same individual, species was set as monoecious and diploid. The allele frequencies were updated during the process in order to account for any changes in inferred sib-ship structure. This avoids collapsing of large families into several small ones due to ignored relatedness [35]. The genotyping errors in marker array technologies are generally very low and were set to 0.0001. Three runs were performed.

### Statistical analysis

#### Additive genetic model

The efficiency of relatedness recovery through sib-ship reconstruction on accuracy of genetic parameters such as additive genetic variance, heritability and genetic correlation was investigated with respect to their standard errors by implementing a linear mixed model within the ASReml-R statistical package [38] as follows:

$$\begin{array}{c} \text{y}=\text{X}\beta +\text{Za}+\text{Zr}+\text{Zr}(\text{s})+\text{e} \end{array}$$

where ***y*** is vector of measurements, ***β*** is vector of fixed effects representing intercept and seed orchard effects, ***a*** is the vector of random breeding values following $\text{var}(a)∼\text{N}(0,A\sigma_{a}^{2})$, where ***A*** is the average numerator relationship matrix [3] developed either from pedigree or on the basis of information from sib-ship reconstruction and $\sigma_{a}^{2}$ is the additive genetic variance, ***r*** is the vector of random replication effect following $\text{var}(r)∼\text{N}(0,I\sigma_{a}^{2})$, where ***I*** is the identity matrix and $\sigma_{r}^{2}$ is the replication variance, ***r(s)*** is the vector of random set nested within replication effects following $\text{var}(r(s))∼\text{N}(0,I\sigma_{\text{r}\left(\text{s}\right)}{}^{2})$, where $\sigma_{\text{r}\left(\text{s}\right)}{}^{2}$ is the variance of set nested within replication effects, ***e*** is the vector of random residuals following $\text{var}(e)∼\text{N}(0,I\sigma_{a}^{2})$, where $\sigma_{e}^{2}$ is the residual variance, ***X*** and ***Z*** are the incidence matrices assigning fixed and random effects to measurements in ***y***.

#### Additive and non-additive genetic model

Since the sib-ship reconstruction produced full-sib relationships (see Results section), it was then possible to construct a dominance relationship matrix and further explore genetic architecture of the investigated traits. In this case, the mixed model was modified as follows:

$$\begin{array}{c} \text{y}=\text{X}\beta +\text{Za}+\text{Zd}+\text{Zr}+\text{Zr}(\text{s})+\text{e} \end{array}$$

where ***d*** is the vector of random dominance effects following $\text{var}(d)∼\text{N}(0,D\sigma_{a}^{2})$, where ***D*** is the dominance relationship matrix created by setting the relationship coefficient of 0.25 between individuals belonging to the same full-sib family, diagonal elements were set to 1 for outbred individuals and 0.5 for selfs [1] and $\sigma_{d}^{2}$ is dominance variance.

#### Multivariate additive genetic model

A bivariate mixed linear model was used to estimate genetic correlations and test its benefit on accuracy of breeding values as follows:

$$\begin{array}{c} \text{Y}=\text{X}\beta +\text{Za}+\text{Zr}+\text{Zr}(\text{s})+\text{e} \end{array}$$

where ***Y*** is matrix of phenotypic measurements, ***a*** is vector of random additive genetic effects following var(***a***)∼N(0, G1), where G1 is variance-covariance structure of additive genetic effects following $\text{G}1=\left[\begin{array}{cc} \sigma_{{\text{a}}_{1}}{}^{2} & \sigma_{{\text{a}}_{1}{\text{a}}_{2}}\\ \sigma_{{\text{a}}_{2}{\text{a}}_{1}} & \sigma_{{\text{a}}_{2}}{}^{2} \end{array}\right]⊗A$, where ⊗ is the Kronecker product, $\sigma_{\text{a}}{{}_{{}_{1}}}^{2}$ and $\sigma_{\text{a}}{{}_{{}_{2}}}^{2}$ are additive genetic variances of the 1^st^ and 2^nd^ trait, $\sigma_{{\text{a}}_{1}{\text{a}}_{2}}$ and $\sigma_{{\text{a}}_{2}{\text{a}}_{1}}$ are additive genetic covariances between the 1^st^ and 2^nd^ trait, ***r*** is vector of random replication effects following var(***r***)∼N(0, G2), where G2 is variance-covariance structure for replication effects following $\text{G}2=\left[\begin{array}{cc} \sigma_{{\text{r}}_{1}}{}^{2} & 0\\ 0 & \sigma_{{\text{r}}_{2}}{}^{2} \end{array}\right]⊗I$, where $\sigma_{\text{r}}{{}_{{}_{1}}}^{2}$ and $\sigma_{\text{r}}{{}_{{}_{2}}}^{2}$ are replication variances for the 1^st^ and 2^nd^ trait, ***r(s)*** is vector of random effect of set nested within replication following var(***r(s)***)∼N(0, G3), where G3 is variance-covariance structure of set nested within replication effects following $\text{G}3=\left[\begin{array}{cc} \sigma_{\text{r}{\left(\text{s}\right)}_{1}}{}^{2} & 0\\ 0 & \sigma_{\text{r}{\left(\text{s}\right)}_{2}}{}^{2} \end{array}\right]⊗I$, where $\sigma_{\text{r}\left(\text{s}\right)}{{}_{{}_{1}}}^{2}$ and $\sigma_{\text{r}\left(\text{s}\right)}{{}_{{}_{2}}}^{2}$ are set within replication variances for the 1^st^ and 2^nd^ trait, ***e*** is a vector of random residual effects following var(***e***)∼N(0, R), where R is a variance-covariance structure for residual effects following $\text{R}\;=\;\left[\begin{array}{ll} \sigma_{{\text{e}}_{1}}{}^{2} & \sigma_{{\text{e}}_{1}{\text{e}}_{2}}\\ \sigma_{{\text{e}}_{2}{\text{e}}_{1}} & \sigma_{{\text{e}}_{2}}{}^{2} \end{array}\right]⊗I$, where $\sigma_{\text{e}}{{}_{{}_{1}}}^{2}$ and $\sigma_{\text{r}}{{}_{{}_{2}}}^{2}$ are residual variances of the 1^st^ and 2^nd^ trait, $\sigma_{{\text{e}}_{1}{\text{e}}_{2}}$ and $\sigma_{{\text{e}}_{2}{\text{e}}_{1}}$ are residual covariances between the 1^st^ and 2^nd^ trait.

#### Genetic parameter estimation

Two heritability estimates were obtained. Narrow sense heritability estimates were obtained using the original pedigree information or information from sib-ship reconstruction, and was estimated as follows:

$$\begin{array}{c} {\hat{h}}^{2}=\frac{{\hat{\sigma }}_{a}^{2}}{{\hat{\sigma }}_{a}^{2}+{\hat{\sigma }}_{e}^{2}}. \end{array}$$

A second estimate was modified to include the estimated dominance variance using the dominance relationship matrix, and based on the reconstructed sib-ship structure as follows:

$$\begin{array}{c} {\hat{h}}^{2}=\frac{{\hat{\sigma }}_{a}^{2}}{{\hat{\sigma }}_{a}^{2}+{\hat{\sigma }}_{d}^{2}+{\hat{\sigma }}_{e}^{2}}. \end{array}$$

Genetic correlations were estimated as the Pearson-product moment correlation as follows:

$$\begin{array}{c} {\hat{r}}_{g}=\frac{{\hat{\sigma }}_{\text{x},\text{y}}}{\sqrt{{\hat{\sigma }}_{x}^{2}{\hat{\sigma }}_{y}^{2}}} \end{array}$$

where ${\hat{\sigma }}_{\text{x},\text{y}}$ is the estimated genetic covariance between traits x and y, ${\hat{\sigma }}_{x}^{2}$ is the estimated additive genetic variance for the x^th^ trait and ${\hat{\sigma }}_{y}^{2}$ is the estimated additive genetic variance for the y^th^ trait. Accuracies of breeding values were estimated as follows:

$$\begin{array}{c} r=\sqrt{1-\frac{PEV}{\left(1+F_{i}\right)\sigma_{a}^{2}}} \end{array}$$

where PEV is prediction error variance and F_i_ is inbreeding coefficient of i^th^ individual [39]. The effect of inbreeding depression on traits’ performance was investigated through comparison of phenotype distributions for outbred individuals vs. selfs. The difference between means was tested with Welch’s t-test [40]. Effective population size of the sample was estimated in terms of the number of founder genome equivalents [41] as follows:

$$\begin{array}{c} N_{\text{ge}}=\frac{1}{{\text{c}}^{\text{T}}\text{Ac}} \end{array}$$

where ***A*** is relationship matrix inferred from either the documented pedigree or the sib-ship reconstruction, ***c*** is vector of contribution where contributions were set equally for each individual as 1/n.

## Results

**Fig 2:**
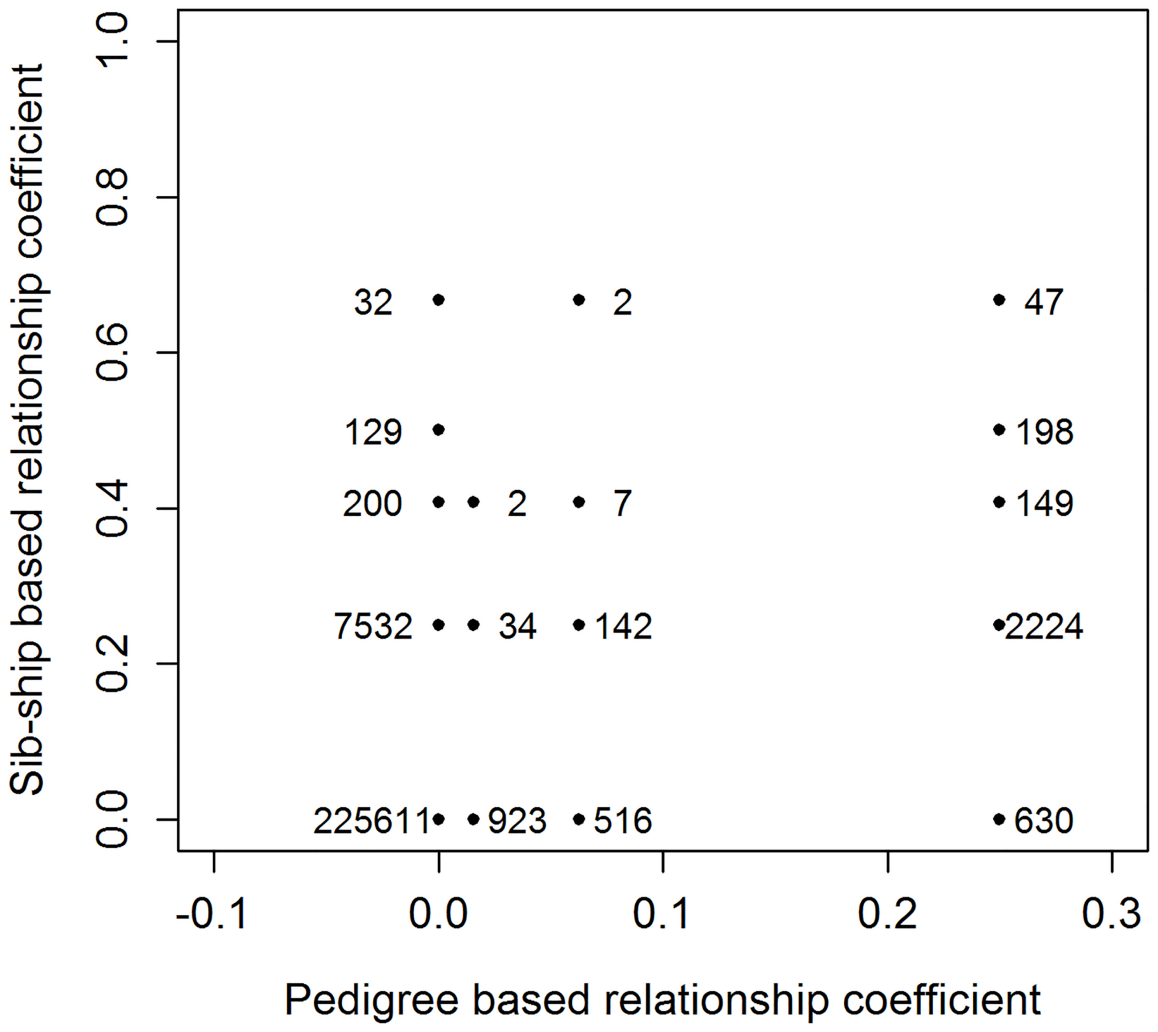
Relatedness agreement. Agreement in relatedness inferred from the pedigree and from sib-ship analysis and number of cases detected are given in the lower triangular relationship matrix (including diagonal) for each scenario.

The EUChip60k [32] provided 4,315 polymorphic SNPs which passed our proposed filter for the *E. nitens* population. From within these, 500 random SNPs were selected for sib-ship analysis. The sib-ship reconstruction was performed in the COLONY package [35] and recovered: 9,932 pairwise half-sib, 334 pairwise full-sib, 368 self half-sib, 81 self full-sib pairs and 27 individuals derived from selfing. This was then compared with 3,248 pairwise half-sib, 684 pairwise first cousins, 959 pairwise second cousins and no selfing based on the documented pedigree information alone (Fig 2). The effective population size in term of founder genome equivalents [41] was estimated to be 196.46 based on documented pedigree and 74.46 based on sib-ship reconstruction information.

**Fig 3:**
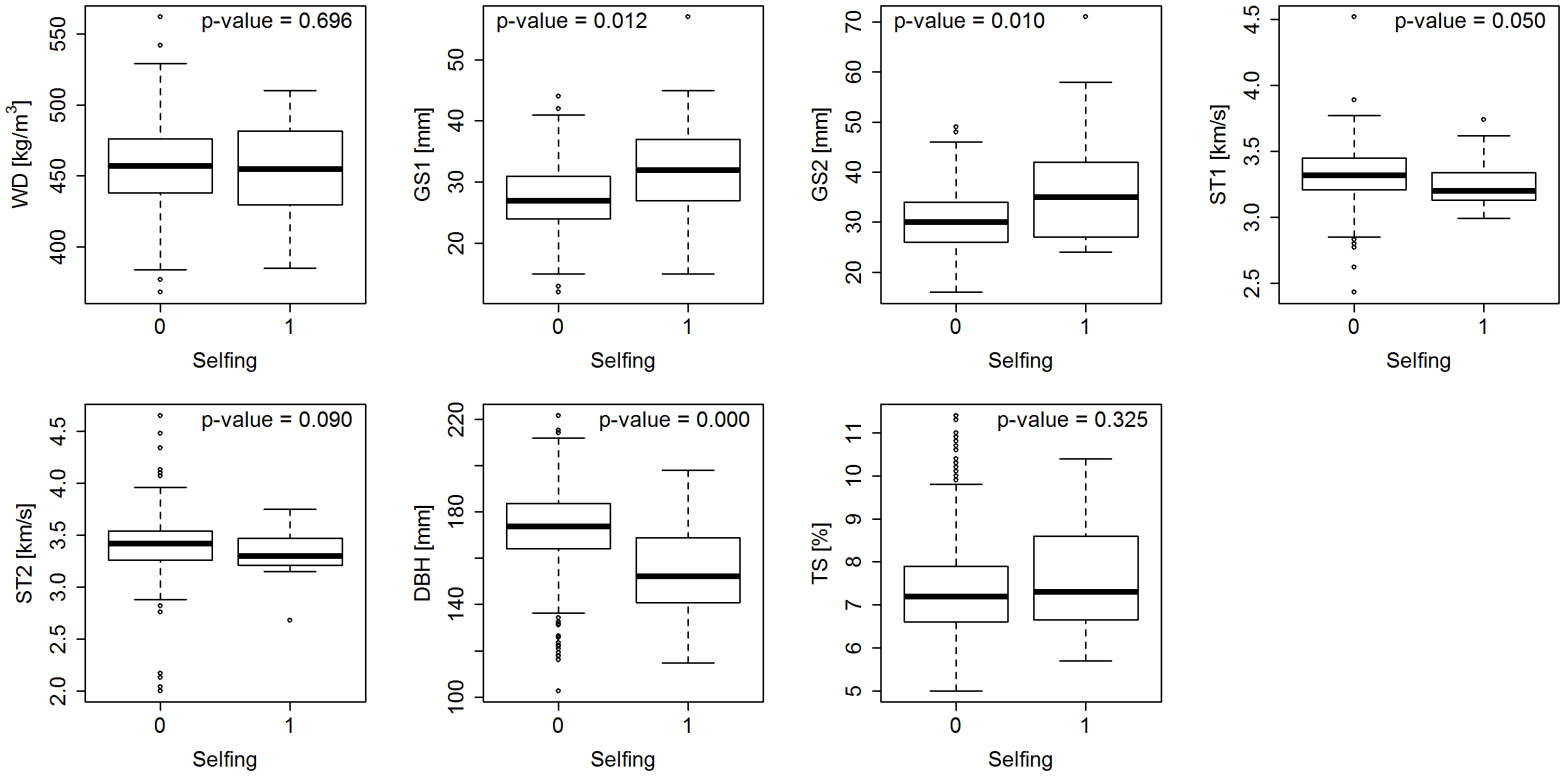
Inbreeding depression effect. Comparison of performance between outbred and inbred individuals after sib-ship reconstruction and Welch t-test p-values; x-axis represents presence (1) or absence (0) of selfing.

We compared the phenotypic performance of selfs and outbred individuals. Since there is the assumption that the selfing reduces genetic variance [42], the statistical significance of differences between means was tested by a Welch’s t-test [40] which is an appropriate alternative when the variance equality assumption is not met. Statistically significant differences were found for DBH, GS1 and GS2 (Fig 3) which indicates that these traits suffer from inbreeding depression.

Estimated heritabilities were low to moderate, ranging from 0.08 (DBH) to 0.45 (WD) and from 0.09 (DBH) to 0.34 (WD) in pedigree and sib-ship reconstruction based analyses. The heritability after sib-ship reconstruction decreased in most of the traits with the largest change from 0.41 to 0.28, observed in TS. By contrast, the heritability of DBH and GS2 increased after sib-ship reconstruction from 0.08 to 0.09 and from 0.18 to 0.22, respectively (Table 1). Across all traits, the implementation of information from sib-ship reconstruction improved the precision of genetic parameters with respect to their standard errors and model fit in terms of AIC. The accuracy of breeding values increased when recovered relatedness was used to estimate genetic parameters with the highest effect reached for traits showing an inbreeding depression effect (DBH, GS1 and GS2 (Fig 3; Table 2)).

**Table 1:** Variance components, heritability, their standard errors and Akaike Information Criterion (AIC) estimated on the basis of pedigree information, sib-ship reconstruction using only additive relationship matrix (Sib-ship A) and sib-ship reconstruction using additive and dominant relationship matrix (Sib-ship AD).

Trait	Model	Additive	Dominance	Replication	Rep(Set)	Error	h^2^	AIC
*WD*	Pedigree	314.9 (100.9)	NA	29.53 (20.08)	38.71 (23.39)	386.4 (88.83)	0.45 (0.133)	5223.1
	Sib-ship A	226.4 (42.33)	NA	33.81 (17.70)	13.69 (17.58)	447.6 (38.68)	0.34 (0.054)	5174.5
	Sib-ship AD	226.4 (42.32)	0.000 (0.000)	33.81 (17.70)	13.69 (17.58)	447.6 (38.68)	0.34 (0.054)	5176.5
*GS*1	Pedigree	7.156 (3.350)	NA	0.243 (0.534)	0.666 (0.829)	21.79 (3.270)	0.25 (0.112)	2845.5
	Sib-ship A	6.676 (1.815)	NA	0.151 (0.527)	0.943 (0.851)	21.60 (1.921)	0.24 (0.060)	2827.8
	Sib-ship AD	6.676 (1.815)	0.000 (0.000)	0.151 (0.527)	0.943 (0.851)	21.60 (1.921)	0.24 (0.060)	2829.8
*GS*2	Pedigree	6.941 (4.110)	NA	2.613 (1.148)	0.000 (0.000)	31.54 (4.199)	0.18 (0.105)	2989.7
	Sib-ship A	8.171 (2.283)	NA	2.502 (1.097)	0.000 (0.000)	29.46 (2.474)	0.22 (0.057)	2968.2
	Sib-ship AD	8.171 (2.492)	0.00 (0.000)	2.502 (1.097)	0.000 (0.000)	29.46 (2.474)	0.22 (0.057)	2970.2
*ST*1	Pedigree	0.009 (0.004)	NA	0.002 (0.001)	0.000 (0.001)	0.025 (0.004)	0.26 (0.111)	-1539.0
	Sib-ship A	0.007 (0.002)	NA	0.001 (0.001)	0.000 (0.001)	0.026 (0.002)	0.23 (0.058)	-1554.5
	Sib-ship AD	0.007 (0.002)	0.004 (0.007)	0.001 (0.001)	0.000 (0.001)	0.022 (0.007)	0.22 (0.061)	-1552.8
*ST*2	Pedigree	0.006 (0.005)	NA	0.003 (0.002)	0.003 (0.002)	0.042 (0.005)	0.13 (0.010)	-1263.6
	Sib-ship A	0.006 (0.003)	NA	0.002 (0.002)	0.004 (0.002)	0.042 (0.003)	0.12 (0.053)	-1269.5
	Sib-ship AD	0.006 (0.003)	0.005 (0.011)	0.002 (0.002)	0.004 (0.002)	0.037 (0.010)	0.12 (0.055)	-1267.8
*DBH*	Pedigree	25.14 (26.17)	NA	0.000 (0.000)	9.031 (7.501)	276.7 (29.44)	0.08 (0.086)	4655.3
	Sib-ship A	28.02 (15.65)	NA	0.000 (0.000)	10.10 (7.61)	271.6 (21.30)	0.09 (0.052)	4652.5
	Sib-ship AD	28.02 (15.65)	0.000 (0.000)	0.000 (0.000)	10.10 (7.608)	271.6 (21.30)	0.09 (0.052)	4654.5
*TS*	Pedigree	0.513 (0.174)	NA	0.065 (0.036)	0.025 (0.035)	0.739 (0.157)	0.41 (0.130)	871.7
	Sib-ship A	0.345 (0.077)	NA	0.048 (0.031)	0.032 (0.035)	0.876 (0.076)	0.28 (0.057)	851.8
	Sib-ship AD	0.325 (0.079)	0.426 (0.222)	0.051 (0.032)	0.031 (0.034)	0.480 (0.205)	0.27 (0.058)	851.2

**Table 2:** Accuracy of breeding values estimated in single trait model, multi-trait model based on documented pedigree and multi-trait model based on information from sib-ship reconstruction.

**Single trait analyses**							
**Model**	**WD**	**GS1**	**GS2**	**ST1**	**ST2**	**DBH**	**TS**
**Pedigree**	0.67	0.53	0.44	0.55	0.39	0.33	0.65
**Sibship—A**	0.70	0.61	0.59	0.60	0.48	0.44	0.66
**Sibship—AD**	0.70	0.61	0.59	0.59	0.46	0.44	0.64
**Multi trait analyses—Pedigree**							
**Model**	**WD**	**GS1**	**GS2**	**ST1**	**ST2**	**DBH**	**TS**
**WD**	X	0.68	0.68	0.69	0.69	0.69	0.68
**GS1**	0.52	X	0.55	0.57	0.52	0.52	0.60
**GS2**	0.45	0.54	X	NA	0.46	0.47	0.54
**ST1**	0.62	0.59	NA	X	0.55	0.56	0.53
**ST2**	0.42	0.41	0.40	0.52	X	0.41	0.39
**DBH**	0.51	0.33	0.33	0.40	0.37	X	0.34
**TS**	0.66	0.69	0.68	0.65	0.65	0.66	X
**Multi trait analyses—Sib-ship reconstruction**							
**Model**	**WD**	**GS1**	**GS2**	**ST1**	**ST2**	**DBH**	**TS**
**WD**	X	0.69	0.70	0.70	0.70	0.70	0.70
**GS1**	0.60	X	0.62	0.61	0.61	0.61	0.65
**GS2**	0.59	0.61	X	0.60	0.59	0.59	0.58
**ST1**	0.63	0.60	0.61	X	0.62	0.62	0.60
**ST2**	0.51	0.50	0.48	0.60	X	0.50	0.48
**DBH**	0.46	0.45	0.48	0.49	0.47	X	0.45
**TS**	0.66	0.69	0.66	0.66	0.66	0.66	X

When a dominance relationship matrix was included in the model, dominance variance was uncovered in ST1, ST2, and TS but was only found statistically significant in the last trait (Table 1). However, the implementation of the dominance matrix did not result in any concurrent improvement in genetic parameters, model fit or breeding values accuracy (Tables 1 and 2).

Multivariate analysis based on pedigree information resulted in only a slight improvement in the accuracy of breeding values compared with results from the univariate analysis (Table 2) and mostly non-significant genetic correlations with five exceptions between GS1 and GS2; ST1 and ST2; ST2 and WD; ST1 and WD; TS and GS1 representing high and significant genetic correlations (Table 3).

**Table 3:** Pairwise genetic correlations and their standard errors estimated based on information from pedigree (above diagonal) and from sib-ship reconstruction (below diagonal).

**Trait**	**WD**	**GS1**	**GS2**	**ST1**	**ST2**	**DBH**	**TS**
**WD**	1	0.14 (0.271)	-0.12 (0.308)	0.79 (0.170)	0.45 (0.306)	0.63 (0.464)	-0.30 (0.216)
**GS1**	0.00 (0.159)	1	0.97 (0.128)	0.66 (0.272)	0.27 (0.420)	-0.20 (0.464)	0.56 (0.265)
**GS2**	-0.06 (0.164)	0.86 (0.089)	1	NA	0.32 (0.456)	-0.21 (0.508)	0.50 (0.308)
**ST1**	0.63 (0.114)	0.20 (0.189)	-0.07 (0.197)	1	0.92 (0.250)	0.39 (0.534)	-0.19 (0.265)
**ST2**	0.55 (0.177)	0.42 (0.234)	-0.03 (0.248)	0.95 (0.125)	1	-0.53 (0.659)	-0.09 (0.360)
**DBH**	0.26 (0.230)	-0.29 (0.248)	-0.53 (0.221)	0.32 (0.290)	0.38 (0.377)	1	-0.08 (0.424)
**TS**	-0.28 (0.137)	0.43 (0.159)	0.24 (0.176)	-0.14 (0.174)	-0.19 (0.232)	-0.05 (0.251)	1

The implementation of information from sib-ship reconstruction in multivariate analysis resulted in mixed results for estimation of genetic correlations, reflecting trends in additive genetic variance. Traits that showed a decrease in additive genetic variances resulted in reduced pairwise genetic correlations and vice versa. The highest improvement in genetic correlation estimated using sib-ship information was reached for traits suffering from inbreeding depression (DBH and GS2) (Table 3). Similarly, the best improvement in breeding values accuracy when using the multivariate model was achieved when traits suffered from inbreeding depression and traits that had high pairwise genetic correlations.

**Fig 4:**
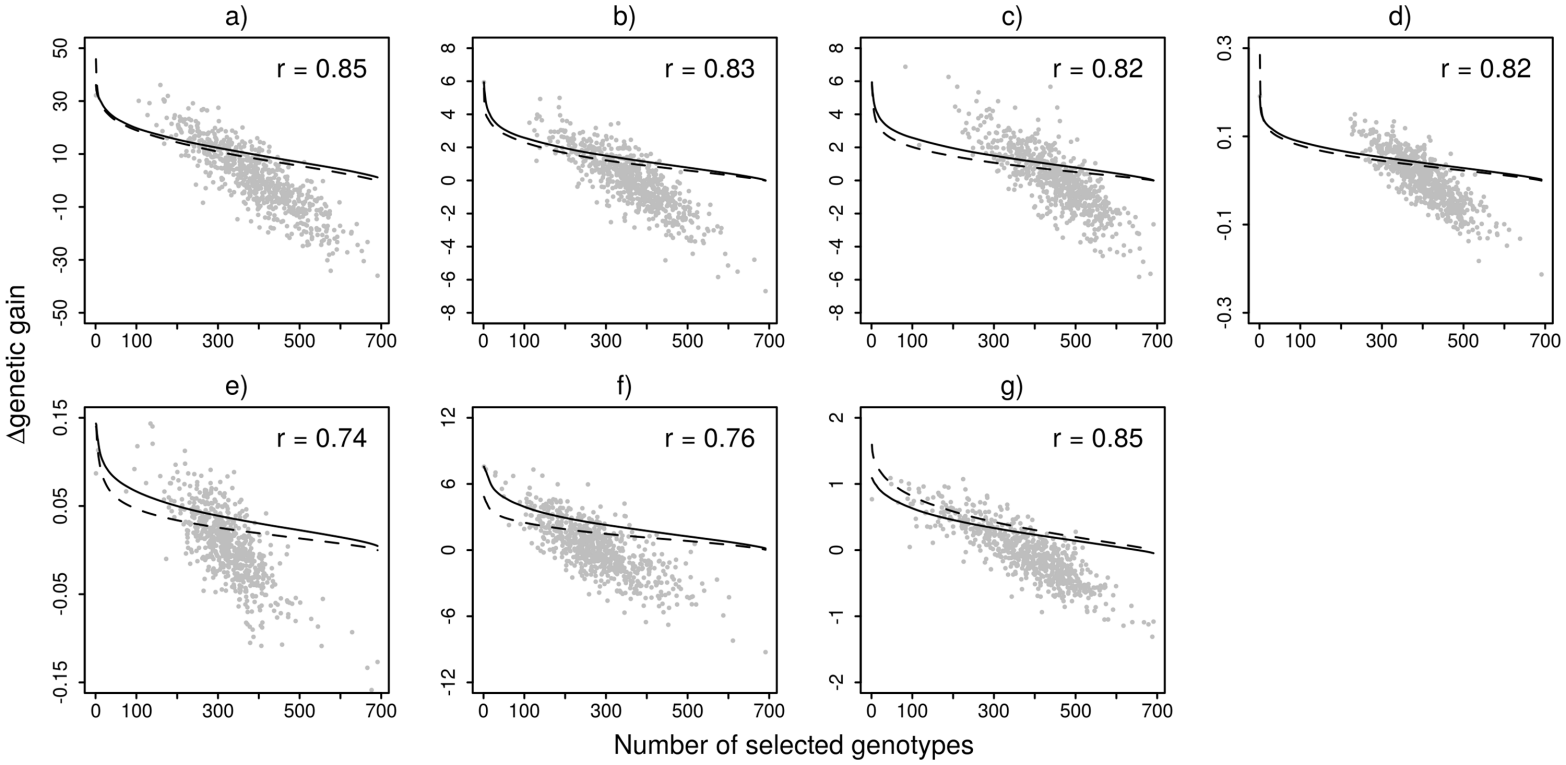
Correspondence of estimated genetic gain and breeding values. Correspondence of genetic gain and breeding values estimated on the basis of information from pedigree (dashed line) or from sib-ship reconstruction (solid line); a) Wood density [kg/m3], b) Growth strain 1.4-3 m log [mm], c) Growth strain 3-6 m log [mm], d) Stiffness 1.4-3 m log [km/s], e) Stiffness 3-6 m log [km/s], f) Diameter at breast height [mm], g) Tangential air-dry shrinkage average 3-6 m log [%].

The effect of sib-ship reconstruction in breeding values ranking was investigated with Pearson product moment correlations as well as with comparison of estimated genetic gains. The correlation between breeding values reached from 0.74 to 0.85 and corresponded to the changes in estimated genetic gain. The highest impact of sib-ship reconstruction was observed in DBH where the response to selection increased for 50% compared to pedigree based analysis (Fig 4). This is most likely the consequence of taking inbreeding depression effect into account through selfing recognition (Fig 3). Similar pattern was observed also in other traits showing signs of inbreeding depression but the effect is most obvious under the strongest selection intensities. The opposite pattern was observed in WD and TS where the reduction in response to selection is connected to decrease in heritability after sib-ship reconstruction (Table 1).

## Discussion

The size, amount and precision of genetic parameter estimates dictates the level of genetic progress in any breeding activity. Heritability, an essential genetic parameter, is heavily influenced by the principle resemblance between relatives [1] and reliable pedigreed field experiments are crucial to perform an accurate genetic evaluation. In forest trees, however the flowering seasonality, inequality between genotype contributions to pollination and labor-intensive nature of performing controlled-pollination at height, prohibits following a prescribed mating design and instead open pollinated experiments are often established [43]. These incomplete open-pollinated pedigrees cause bias in genetic parameter estimates and prohibits full exploration of genetic variance within the selection procedure.

Pedigree reconstruction [19] has been proven as an efficient tool to convert open pollinated tests with an incomplete pedigree into pseudo-family tests with complete pedigree information [8, 10, 23, 25, 44]. However, the method suffers when a large proportion of the fathers come from exogenous resources outside of the parental population (pollen contamination). Pollen contamination has the potential to reduce the impact of parental reconstruction on the precision of genetic parameters. Proper pre-selection of candidates for genotyping is required to reduce contamination rate [45, 46]. Alternatively, use of a polycross can be considered a viable strategy to reduce pollen contamination to a level efficient for pedigree reconstruction [23].

Our study investigates a third generation of breeding population within the New Zealand *E. nitens* breeding program, where only the maternal side of the pedigree was tracked across all generations. Sib-ship reconstruction recovered the main portion of the pedigree-based half-sib relationships and also identified pedigree errors. Nevertheless, the methodology can only recognize major classes of relatedness such as unrelated, half-sib and full-sib pairs [35], which is not always the case in advanced generation breeding populations. This resulted in the disappearance of the low level relatedness class of first and second cousins and moved them to unrelated (in most cases) or to the greater degree of relatedness such as half- and full-sibs (Fig 2). Therefore, either scenario does not provide the true relatedness but the sib-ship reconstruction creates the most likely case. When genetic markers are available for both parental and offspring population, the parentage rather than sib-ship reconstruction should be considered in the population with a complex pattern of relatedness. In addition, the number of markers used in pedigree reconstruction effects the efficiency of the assignment rate. Huisman [36] found at least 200 SNPs should be used in parentage analysis to reach over 99% of assignment rate. However, the inability to recover the low level relatedness classes did not appear to harm downstream analyses and the scenarios based on information from sib-ship reconstruction resulted in an improved model fit in terms of AIC (Table 1). Any of the above mentioned procedure does not allow the effective tracking of Mendelian sampling to get closer to true relatedness. Many genetic markers would be needed to make reliable inferences about the Mendelian segregation [47, 48]. Cappa et al [28] tested different types of molecular markers to construct relatedness and found the advantage of co-dominant (SSR, SNP) over dominant (DArT) markers in ability to capture Mendelian segregation. Bush et al [49] proposed family-wise correction to reflect differences in genetic similarities within each family in mixed mating system based on sparse set of genetic markers. However, we found this procedure difficult to apply in advanced breeding populations through more complex relatedness which could result in loss of semi-positive definity of the resulting relationship matrix, a requirement of mixed linear models.

Open pollination lacks inbreeding control and our sib-ship reconstruction recovered 27 individuals (4%) derived from selfing events, which largely contributed to a decrease in effective population size from 196.46 to 74.46. Selection of such individuals for the next generation of the breeding population would result in an undesirable increase in inbreeding level when no control over the paternal contribution is performed. This could potentially result in a considerable decrease of effective population size, needed for the long-term sustainability in genetic progress. Additionally, higher inbreeding levels may cause inbreeding depression, which in some populations/traits causes inferior phenotypic expression [50–53].

We investigated the phenotypic performance of individuals derived from selfing compared with individuals derived from outcrossing (Fig 3). The average performance of selfs compared with outcrossed progeny was inferior in DBH, GS1 and GS2, but did not affect the performance in WD and TS. These results are consistent with a previous study in *E. nitens* [54] which found the effect of inbreeding depression present in growth traits but not in wood density. The presence of inbreeding depression in growth traits was observed even in other *Eucalyptus* species [49]. However the effect of inbreeding depression is issue not only in term of inferior productivity but also aborted embryos and the survival rate of seedlings [55] which makes nursery operation less effective. Therefore, the recognition of selfs can be especially important in multi-trait selection when both types of traits are considered in construction of selection indexes.

Most of the traits showed a reduction or no change in heritability estimates and no change in accuracy of breeding values when sib-ship was used, which corresponds with findings in Vidal et al. [23]. Other studies [8, 9, 27] found a large reduction in additive genetic variance and heritability after partial pedigree reconstruction but considerable improvement in model fit. They attributed those results to better dissection of genetic and micro-site environmental variance when multiple trees are tested at each plot [56]. The opposite pattern was observed in DBH, GS1 and GS2 where heritability and breeding values accuracy (Tables 1 and 2) as well as expected response to selection (Fig 4) increased considerably. These three traits highlight the inferior performance of selfs compared with outbred individuals (Fig 3), which indicates a deleterious effect of inbreeding depression. Doerksen and Herbinger [10] found a decrease in genetic parameters as additive genetic variance and heritability in population showing 3% of selfs, but they did not find any evidence for the presence of inbreeding depression in their traits. Therefore, the unrecognized selfing in documented pedigree analysis appears to downwardly bias additive genetic variance and heritability estimates in traits showing signs of inbreeding depression.

The recovery of full-sib families allowed us to construct a dominance relationship matrix, which was implemented in the mixed model to investigate non-additive genetic variance. Our analysis identified dominance variance in ST1, ST2, and TS but it was significant only in the last trait. The implementation of non-additive relationship matrices, especially dominance, improved the accuracy of genetic parameters in previous studies [57–59]. However, we did not find any concurrent improvement in model fit or precision of genetic parameters when the dominance relationship matrix was included in the model. The lack of observable improvement when implementing a dominance matrix is likely caused by the absence of functional dominance in the genetic architecture of the studied trait [57] or by the lack of higher class of relatedness in the population needed for reliable exploration of non-additive genetic component. The fitting of a non-additive effect through the specific relationship matrices allows the estimation of genotypic values commonly used in clonal forestry [56, 60, 61]. Along the additive and dominance relationship matrix, the epistatic relationship matrices can be estimated as their Hadamard products [62]. However, they were ignored in this study due to lack of relatedness in the population and small sample size. Additionally, the information from non-additive genetic components can be useful in decision about mating allocation to increase frequency in favorite alleles [59].

Multivariate analyses can further improve the precision of genetic parameters through genetic correlations [63, 64]. Our results showed only small improvements in the accuracy of breeding values in multivariate analysis which was probably connected to the mostly non-significant estimates of genetic correlations and lack of data. The substantial increase in the accuracy of breeding values was achieved with ST2 and GS2 likely due to high and significant genetic correlations with ST1 and GS1, respectively. Therefore, the genetic correlation between traits must be strong in order to achieve any substantial improvement, otherwise the multivariate analysis can reduce accuracies [64].

The precision of genetic correlations and the accuracy of breeding value estimates showed large improvement when information from sib-ship reconstruction was implemented. However, many genetic correlation estimates decreased and thus remained non-significant. Generally, about 100 families should be tested to obtain reliable and significant genetic correlations [65]. The genetic correlation between DBH and GS2 where intermediate, not significant negative correlation became after sib-ship reconstruction, high negative and significant is the exception. Therefore, the pattern in estimated genetic correlations reflects the pattern in additive genetic variances (Tables 1 and 3), and we observed the benefit of sib-ship reconstruction only in traits showing signs of inbreeding depression (Fig 3).

Overall, the correspondence between breeding values produced in pedigree based and sib-ship reconstruction based model were between 0.74 and 0.85 (Fig 4) which is rather low compared with the 0.96–0.97 range reported in Vidal et al. [23]. Such discrepancy between breeding values results in changes in individual’s ranking and sib-ship reconstruction provides tool to achieve improved identification of the genetically superior individuals. Additionally, obtaining more accurate relatedness structure in the population enables more efficient management of genetic diversity. Hidden relatedness and pedigree errors also have the potential to downwardly bias the predictive accuracy in genomic selection evaluation based on the correlation of estimated breeding values and genomic breeding values when pedigree reconstruction is not performed before genomic evaluation.

In addition, the estimated response to selection rapidly changed with change in heritability between investigated strategies. For traits suffering from inbreeding depression such as DBH, GS1 and GS2, the estimated genetic gain based on the original pedigree was largely underestimated. This was especially the case for DBH where the genetic gain when using sib-ship reconstruction increased for 50% compared with the scenario based on the original pedigree (Fig 4). In contrast, the traits showing a decrease in heritability due to hidden relatedness (WD, ST1, TS), showed an upwardly biased estimate of genetic gain. Such bias in estimated genetic gain can produce misleading information about the performance of breeding program and mismanagement of selection strategies.

## Conclusion

Our analyses showed benefits from sib-ship reconstruction by identification of selfs to investigate the possible detrimental effect of inbreeding depression found in three studied traits. This allowed us to understand different patterns in genetic parameter changes when information from sib-ship reconstruction was implemented. Despite only rough inference about relatedness in advanced breeding populations due to an inability to recover the low classes of relatedness, sib-ship reconstruction provides a useful tool to recover major categories of relatedness where parental genomic information is missing. The technique will enable more effective management of genetic diversity and provide more precise estimates of genetic parameters compared with the previously incomplete documented pedigree.

We found the implementation of sib-ship reconstruction resulted in higher heritability and genetic correlation estimates and improved accuracy of breeding values in traits showing signs of inbreeding depression. In traits free of inbreeding depression, the impact of sib-ship reconstruction was a decrease in heritability and only a negligible improvement in breeding values accuracy. We found lower correspondence between breeding values estimated from pedigree and sib-ship reconstruction, which can potentially bias the predictive ability in a genomic evaluation and prior pedigree reconstruction is recommended. Sib-ship reconstruction will be very useful implementation tool for breeders to ensure sufficient genetic diversity is present through inbreeding identification and eliminate selfs from selection in open-pollinated programs.
